# Evaluation of Hydrogenation Kinetics and Life Cycle Assessment on Mg_2_NiH_x_–CaO Composites

**DOI:** 10.3390/ma14112848

**Published:** 2021-05-26

**Authors:** Hyo-Won Shin, June-Hyeon Hwang, Eun-A Kim, Tae-Whan Hong

**Affiliations:** Department of Materials Science and Engineering, College of Engineering, Korea National University of Transportation, Chungju 27469, Korea; whun2288@ut.ac.kr (H.-W.S.); hj930403@ut.ac.kr (J.-H.H.); euna_0106@ut.ac.kr (E.-A.K.)

**Keywords:** hydrogen storage, kinetics, material life cycle assessment, Eco-Indicator 99’, CML 2001

## Abstract

Magnesium-based alloys are attractive as hydrogen storage materials due to their lightweight and high absorption, but their high operating temperatures and very slow kinetics are obstacles to practical applications. Therefore, the effect of CaO has improved the hydrogenation kinetics and slowed down the degradation. The Mg_2_NiH_x_–CaO composites were prepared by hydrogen-induced mechanical alloying (HIMA). Hydrogenation kinetics was performed by using an Automatic PCT Measuring System and evaluated in the temperature range of 423, 523, and 623 K. As a result of calculating the hydrogen absorption amounts through the hydrogenation kinetics curve, they were calculated as about 0.52 wt%, 1.21 wt%, and 1.59 wt% (Mg_2_NiH_x_–10 wt% CaO). In this study, the material environmental aspects of Mg_2_NiH_x_–CaO composites were investigated through life cycle assessment (LCA). LCA was performed analyzing the environmental impact characteristics of the manufacturing process by using Gabi software and the Eco-Indicator 99’ and Centrum voor Milieuweten schappen (CML 2001) methodology. As a result, the contents of global warming potential (GWP) and fossil fuels were found to have a higher impact than other impact categories.

## 1. Introduction

Recently, due to greenhouse gas emissions and global warming problems, the need to develop a new and renewable energy source that can replace fossil fuels has increased, and hydrogen, a clean energy media, has attracted attention [1]. Hydrogen is known as a clean energy media because, unlike fossil fuels, it cannot be used directly in nature, and it is produced from primary energy sources to produce energy through internal combustion engines or fuel cells, and only water is produced as a by-product [2,3]. Accordingly, in January 2019, the government announced the “Hydrogen Economy Revitalization Roadmap” to realize a zero-carbon society and lead the transformation of the energy paradigm by utilizing hydrogen with a high energy storage density (33.3 kWh/kg H_2_). To realize these hydrogen economies, technological innovation in the production, storage, transportation, and utilization of hydrogen are necessary. However, in the case of hydrogen storage technology, which is essential for safely storing and transporting hydrogen, secure technology is urgently needed because investment is not made relatively [4].

Among the hydrogen storage methods, metal hydrides belonging to hydrogen storage alloys are produced by the reaction between metal and hydrogen, and the metal adsorbs hydrogen gas, and when heated again, it releases hydrogen. The reaction in which hydrogen reacts with a metal to form metal hydride (MH) is an exothermic reaction [5]. In particular, Mg-based hydride has the advantages of having high hydrogen storage, a low cost, and being lightweight. The surface is thermodynamically stable and has a very slow hydrogenation reaction rate [6,7,8].

To improve these obstacles, studies on the catalytic effect were conducted by adding a transition metal, and in the case of metal hydride (Mg_2_NiH_4_), in order to change the thermodynamic stability, there have been studies on improving the storage and release characteristics of hydrogen by substituting various elements such as Ca and rare earth metals for Mg and Ni in the Mg_2_Ni alloy [9]. In this study, Mg_2_NiH_x_–CaO composites were prepared by adding CaO to Mg_2_NiH_x_ using hydrogen-induced mechanical alloying (HIMA). The effect of catalyst and oxidation resistance was investigated by paying attention to the hydrogenation behavior according to the added alkaline earth metal oxide.

Life cycle assessment (LCA) is an input and output to assess the environmental impact of a product or service throughout the entire process (raw material collection, product production, use, disposal), that is, resource depletion due to inputs, and environmental impacts caused by discharges. It can be said that it is a process of reviewing alternatives to improve environmental performance by preparing a quantitative data list of the data, evaluating the environmental impact [10]. In addition, as it forms the technical basis of the ISO 14000 series, it can be said to be an internationally important technique [11]. These results enable a fair comparison of products or processes and can also contribute to product design that minimizes environmental impact. Problems related to toxic emissions or waste can be solved by replacing materials or processes, as well as effects related to raw materials and energy consumed [12]. While LCA is a valuable way to measure environmental loads such as “cradle-to-grave”, it has limitations when it comes to obtaining and evaluating data about the entire production process. LCA uses an internationally standardized methodological framework for analyzing the environmental impacts associated with the life cycle phases of products, processes, or activities over their entire life, typically from cradle-to-grave [13]. All products are made from materials, and one material is made using different technologies or used in different products [14]. Material life cycle assessment literally provides an important tool for material research as an environmental evaluation method that focuses on materials rather than processes. There is a case in which the potential environmental impact of recycling of indium tin oxide (ITO) transparent electrodes separated from the display panel has been studied with this material-focused environmental evaluation method [15]. Therefore, in this study, LCA was carried out to confirm that the hydrogenation kinetics of the Mg_2_NiH_x_–CaO composites were improved and to evaluate the potential environmental impact of the material.

## 2. Experimental Procedure

### 2.1. Specimen Preparation and Characterization

Mg (Sigma-Aldrich, St. Louis, MO, USA, 98%) and Ni (Sigma-Aldrich, St. Louis, MO, USA, 99.7%) powder was charged into a 1/2-inch STS304 container. At this time, the weight ratio of Mg and Ni powder was designed as 45:55 with reference to the Mg–Ni binary phase diagram. After making a vacuum up to 5 × 10^−2^ Torr using a rotary pump, hydrogen of 99.9999% purity was applied to a pressure of 3.0 MPa and alloyed for 96 h at a rotational speed of 200 rpm using a planetary ball mill (Pulverisette-5, FRITSCH Co., Idar-Oberstein, Germany), which is a hydrogen-induced mechanical alloying method. At this time, the ball to chips weight ratio (BCR) of 1/2-inch chrome steel balls and magnesium chips was set to 66:1 with reference to the preceding paper [16]. Then, the prepared powder and 5, 10 wt% CaO (Sigma-Aldrich, 99.7%) in the form of powder were charged into a container and alloyed for 24 h at a rotation speed of 200 rpm under the same conditions.

For the metallurgical characterization of the sample prepared through the alloying process, the crystal structure and phase of the sample were characterized using X-ray diffraction (XRD) analysis (D8 Advance, Bruker, Billerica, MA, USA), which was performed using a Cu Kα radiation of 1.5405 Å (scanning speed: 3 deg/min, scanning angle: 20–80°). In order to observe the surface shape and particle size of the sample according to the alloying time, it was observed using scanning electron microscopy (SEM) (FEI Quanta 400, FEI, Hillsboro, OR, USA), and Brunauer–Emmett–Teller (BET) surface analysis (Micromeritics-3-Flex, Heidelberg, Germany) was used to measure the particle specific surface area, which has a large influence on hydrogen diffusion. After that, the dehydrogenation activation energy was measured by drawing an Arrhenius plot through the dehydrogenation temperature. In addition, Sivert’s type automatic PCT (pressure–composition–temperature) method, an automated volumetric measurement method, was used to measure the hydrogenation kinetics, and the hydrogen absorption reaction rate was evaluated at a temperature range of 423, 523, and 623 K for 1 h by applying a constant hydrogen pressure of 3.0 MPa.

### 2.2. Life Cycle Assessment (LCA)

The LCA is used to analyze the environmental impact of a product and can provide information on the stages of a product’s life cycle [17]. LCA has an ISO standardization method (ISO 14040 2006; ISO 14044 2006), an LCA consists of four categories: (1) goal and scope definition, (2) life cycle inventory analysis, (3) impact assessment and (4) interpretation of results [18,19]. The quality of the LCA depends on the exact description of the production process to be analyzed. To know where each phase of the life cycle begins and ends properly, you need to collect and interpret its process data. The study used the Centrum voor Milieuweten schappen (CML 2001), a combined lifecycle impact assessment method developed by the University of Leiden to determine the environmental performance of the process under study. The CML method defines several impact categories for emissions and resource consumption as problem-oriented (midpoint).

#### 2.2.1. Goal and Scope

The goal and scope outline the material to be studied, assess the environmental impact categories, and analyze the resulting limitations or assumptions. First of all, it is very important to first establish the decisions to be presented by the evaluation for material selection [20]. The life cycle inventory (LCI) consists of identifications of all unit processes, product-related flows [13]. Additionally, the methodology adopted for LCA analysis in this study complies with the following standards [21]:

ISO 14040, 14044: 2006—Environmental Management—Life Cycle Assessment—Requirements and Guidelines [18,19].

In this study, a cradle-to-gate approach of LCA was applied to assess Mg_2_NiH_x_–5, 10 wt% CaO composites that were manufactured and characterized, and an environmental assessment was carried out throughout the disposal process. The goal is to identify whether hydrogenation kinetics is improved by adding CaO in the synthesis process of Mg_2_NiH_x_–CaO composites, and quantify the resulting environmental load, and analyze the environmental characteristics.

**Figure 1:**
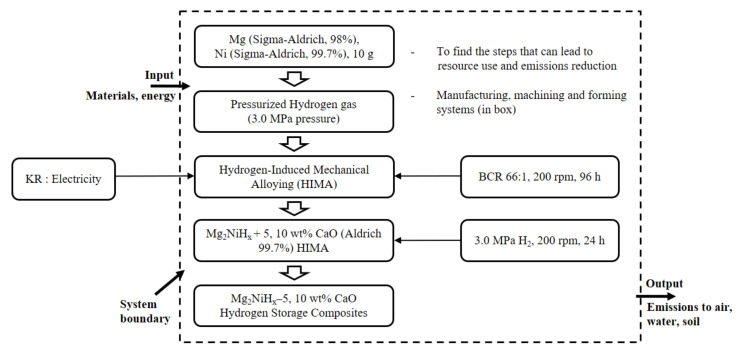
Process flow diagram for Mg_2_NiH_x_–5, 10 wt% CaO synthesis and analyses.

The function of the prepared Mg_2_NiH_x_–CaO composites are the hydrogenation kinetics, and the functional unit, which is a unit representing the function, is set as hydrogen content (wt%), and the reference flow that satisfies the functional unit is 10 g of powder. Figure 1 is composed of a manufacturing step, a characteristic evaluation step, and a disposal step of the Mg_2_NiH_x_–CaO composites in the LCA. The raw material category includes Mg_2_Ni and CaO. The energy category includes electricity used in the manufacturing and characterization stages, and air emissions are carbon monoxide (CO), carbon dioxide (CO_2_), sulfur oxides (SO_x_), nitrogen oxides (NO_x_), and dust generated throughout the process. Data quality requirements were established by dividing the technical, temporal and regional scope into manufacturing, characterization, and disposal stages.

#### 2.2.2. Impact Assessment Categories and Environmental Impact Methodology

In this study, the end-point concept CML 2001 methodology and Eco-Indicato’99 (EI99) methodology developed by Nederland and pre-consulting organizations were used. In this study, the CML 2001 and Eco-Indicator 99’ (EI99) methodology with an end-point concept developed by a Dutch pre-consulting institution were used, and these are shown in Table 1 and Table 2. The software Gabi 6 (Sphera, Stuttgart, AR, USA) was used to perform an environmental impact assessment on the process of synthesizing the composites. The EI99 methodology considers three damage categories: human health, ecosystem health, and resources, of which, the following types of damage are sorted into: carcinogenic, respiratory effects, climate change, radioactivity, ozone layer, ecotoxicity, acidification, land use, resource, and fuel replenishment. As an indicator, in the human health category, the index of the endpoint level is derived using the disability-adjusted life years (DALY) as an indicator. It is expressed as the probability (PDF × m^2^ × yr) of the potential disappearance of species per area (m^2^), and in the resource depletion category, the surplus energy input to harvest 1 kg of resources is selected as an indicator [22].

## 3. Results and Discussion

### 3.1. Evaluation of the Synthesized Composites

**Figure 2:**
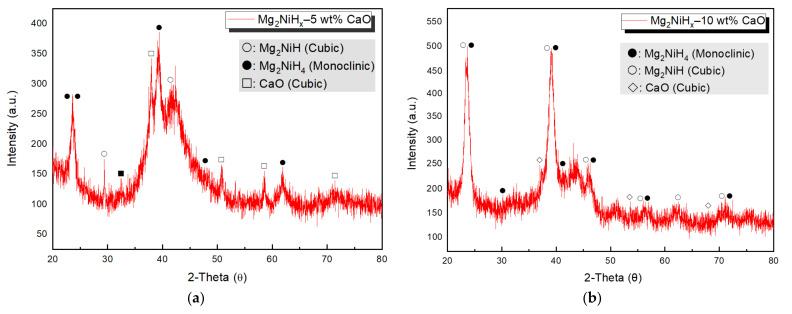
(**a**) Mg_2_NiH_x_–5 wt% CaO (reprinted with permission from. [23]. Copyright 2021. Copyright Shin, H.-W.), (**b**) Mg_2_NiH_x_–10 wt% CaO composites X-ray diffraction (XRD) image.

Figure 2 shows the XRD pattern of the Mg_2_NiH_x_–10 wt% CaO composites, which was ball-milled for 96 h in a hydrogen atmosphere to prepare Mg_2_NiH_x_, and then ball-milled for an additional 24 h by adding 5, 10 wt% CaO. As a result of the analysis, clear peaks of magnesium hydride and calcium oxide appeared, and Mg_2_NiH, Mg_2_NiH_4_, and CaO peaks were identified through the International Centre for Diffraction Data (ICDD). Additionally, Mg_2_NiH_4_ has a monoclinic structure, and Mg_2_NiH and CaO have a cubic structure. 

**Figure 3:**
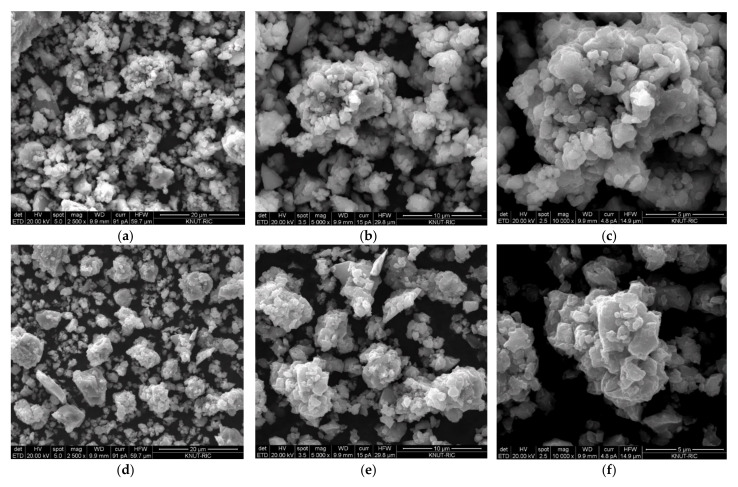
Mg_2_NiH_x_–5 wt% CaO (reprinted with permission from [23]. Copyright 2021. Copyright Shin, H.-W.). SEM morphologies: (**a**) ×2500, (**b**) ×5000, (**c**) ×10,000, Mg_2_NiH_x_–10 wt% CaO SEM morphologies: (**d**) ×2500, (**e**) ×5000, (**f**) ×10,000.

Figure 3 is a scanning electron microscope (SEM) surface shape observation photograph of Mg_2_NiH_x_–CaO composites. Particle sizes vary from 1 to 10 μm and a tendency to clumping has been observed due to the many nano-sized particles and the grinding process. It was pointed out that this cluster formation and irregular shape of the particle size distribution are evidence of the milling effect [24]. According to Huang et al., who studied the relationship between particle size and hydrogen diffusion, nano-sized particles and an increase in specific surface area promoted hydrogen absorption and desorption [25]. As a result of comparing the Mg_2_NiH_x_–5 wt% CaO and Mg_2_NiH_x_–10 wt% CaO composites, it can be seen that the particle size decreases as CaO is added. As the particle size decreases, the diffusion length of hydrogen decreases and the reaction surface area increases, so it is considered to be easier for hydrogen absorption and desorption.

**Figure 4:**
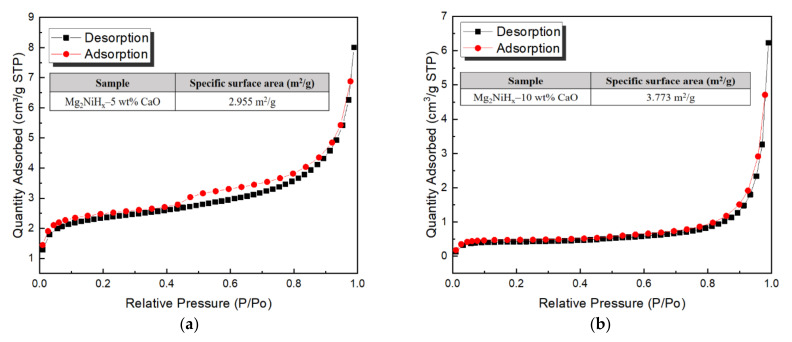
Bruner–Emmett–Teller surface area analysis results for (**a**) Mg_2_NiH_x_–5 wt% CaO (reprinted with permission from [23]. Copyright 2021 Copyright Shin, H.-W.), (**b**) Mg_2_NiH_x_–10 wt% CaO.

Figure 4 is the result of specific surface area analysis (SSA) measuring the nitrogen absorption and desorption behavior of Mg_2_NiH_x_–CaO composites, and SSA is calculated as 2.955 m^2^/g (Mg_2_NiH_x_–5 wt% CaO [23]), 3.773 m^2^/g (Mg_2_NiH_x_–10 wt% CaO). Increasing the SSA of nanoparticles promote absorption and desorption of hydrogen but increases the alloying time and decreases the size of the particles, increasing the formation of nano and amorphous phases [26]. Comparing the Mg_2_NiH_x_–5, 10 wt% CaO composites, the specific surface area increases with the addition of CaO, and thus the absorption and desorption behavior of hydrogen is expected to be advantageous.

**Figure 5:**
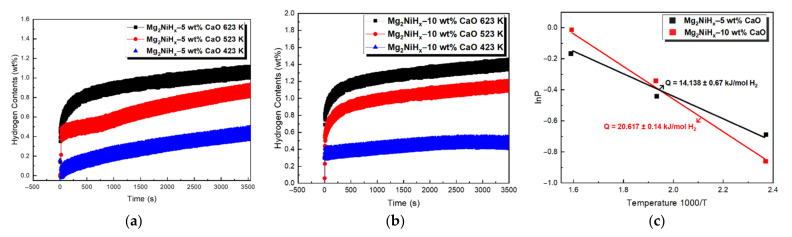
Hydrogen absorption kinetics of (**a**) Mg_2_NiH_x_–5 wt% CaO (reprinted with permission from [23]. Copyright 2021 Copyright Shin, H.-W.), (**b**) Mg_2_NiH_x_–10 wt% CaO, (**c**) calculation of van’t Hoff plots on Mg_2_NiH_x_–5 wt% CaO (reprinted with permission from [23]. Copyright 2021 Copyright Shin, H.-W.) and Mg_2_NiH_x_–10 wt% CaO.

Figure 5a is the result of measuring the hydrogen absorption reaction kinetics of Mg_2_NiH_x_–10 wt% CaO composites under each temperature (423, 523, 623 K) condition. After the hydrogen pressure was kept constant at 3.0 MPa, the change in hydrogen absorption with time for 1 h was investigated. As a result, hydrogen absorption was highest at 623 K and lowest at 423 K. When the effective hydrogen storage amount was calculated through the hydrogenation kinetics curve, Mg_2_NiH_x_–5 wt% CaO at 423, 523, and 623 K temperatures were 0.51 wt%, 0.93 wt%, and 1.12 wt%. Mg_2_NiH_x_–10 wt% CaO showed 0.52 wt%, 1.21 wt%, 1.59 wt% of hydrogen absorption, through which it was confirmed that the hydrogenation reaction rate increased as CaO was added. Figure 5b is the result calculated through the van’ t Hoff equation from the result of the hydrogen absorption reaction rate. The absorption enthalpy (ΔH) of the Mg_2_NiH_x_–5 wt% CaO was calculated as a value of 14.138 ± 0.67 kJ/mol [23], and the Mg_2_NiH_x_–10 wt% CaO showed a relatively high value of 20.617 ± 0.14 kJ/mol. Therefore, Mg_2_NiH_x_–10 wt% CaO composites exhibited superior kinetics characteristics compared to Mg_2_NiH_x_–5 wt% CaO due to the high heat of reaction.

### 3.2. Life Cycle Assessment on Composites Prepared

The LCA process uses classification, characterization, and normalization. The environmental impact is then derived according to this order, and major issues are then identified, based on this. In our work, the classification involved 10 impact categories, which included abiotic resource depletion (ARD), global warming potential (GWP), stratospheric ozone depletion potential (ODP), acidification potential (ACP), and eutrophication potential (EUP), ecotoxicity potential (ETP), and human toxicity potential (HTP). Among these, ecological toxicity included fresh-water aquatic ecotoxicity potential (FAETP), marine aquatic ecotoxicity potential (MAETP), and terrestrial ecotoxicity potential (TETP). In addition, 11 impact categories are included in human health, ecosystem quality, and resources [27].

**Figure 6:**
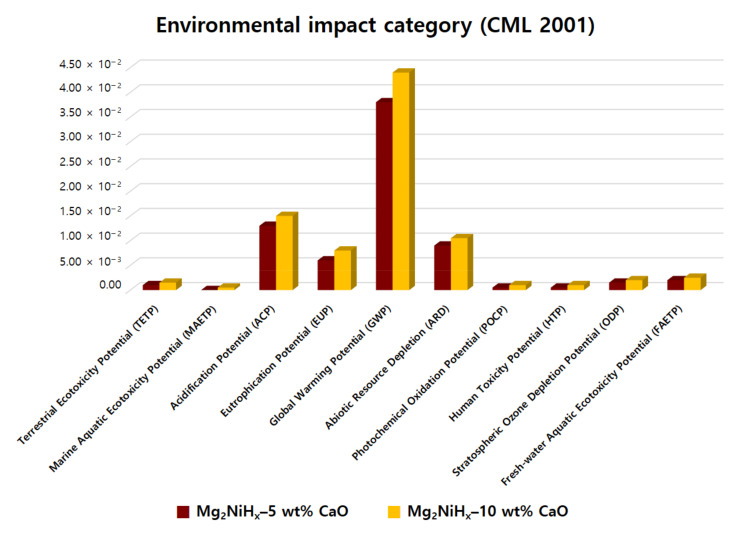
Normalization of Mg_2_NiH_x_–5 wt% CaO (reprinted with permission from [28]. Copyright 2021 Copyright Shin, H. W) and Mg_2_NiH_x_–10 wt% CaO composites by environmental impact category (CML 2001).

In Figure 6, the normalization result of applying CML 2001 to the Mg_2_NiH_x_–CaO composite material is plotted as one graph through the comparison target. As a result, it was confirmed that both Mg_2_NiH_x_–5, 10 wt% CaO composites showed the highest GWP value, followed by ACP and ARD. As can be seen from the graph, Mg_2_NiH_x_–10 wt% CaO overall showed a higher value than Mg_2_NiH_x_–5 wt% CaO, which is considered to be the effect of CaO addition. In addition, the highest GWP value appears to be the use of electricity through multiple mechanical alloying (MA) processes. Therefore, it is necessary to study a process that can be synthesized by performing a single MA process when manufacturing Mg_2_NiH_x_–CaO composite materials, and to find a way to lower the global warming index by reducing electricity consumption.

**Figure 7:**
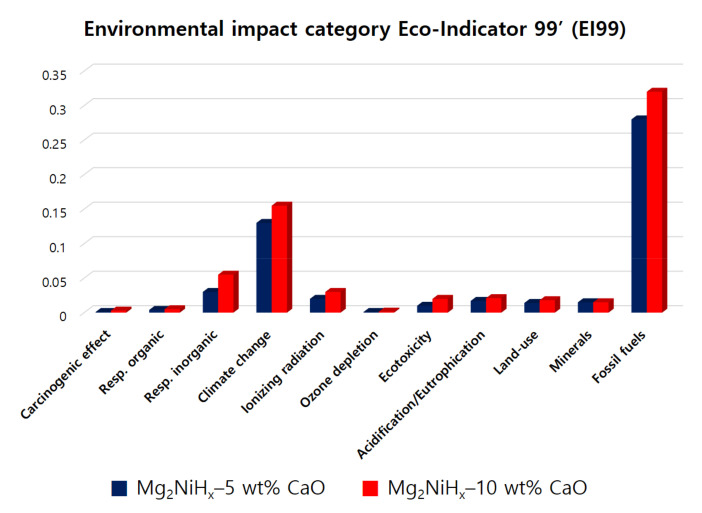
Normalization of Mg_2_NiH_x_–5 wt% CaO (reprinted with permission from [28]. Copyright 2021 Copyright Shin, H.-W.) and Mg_2_NiH_x_–10 wt% CaO compo-sites by environmental impact category Eco-Indicator 99’ (EI99).

Figure 7 shows the Mg_2_NiH_x_–CaO composites as a graph using the Eco-Indicator 99’ (EI99) methodology. As a result of the graph, fossil fuels were the largest. In addition, the impact categories were measured in the order of climate change and respiratory. In particular, Mg_2_NiH_x_–10 wt% CaO shows the greatest difference between fossil fuels and climate change values when compared to Mg_2_NiH_x_–5 wt% CaO, which appears to be highly related to global warming, similar to the previous CML 2001 methodology. In view of the fact that the remaining environmental impact figures show almost similar values, it is judged that the additional amount of 5 wt% CaO does not have a significant effect. As mentioned above, efforts are required to minimize unnecessary energy consumption during the manufacturing process and to reduce the use of electricity as much as possible to reduce a large amount of environmental load.

**Figure 8:**
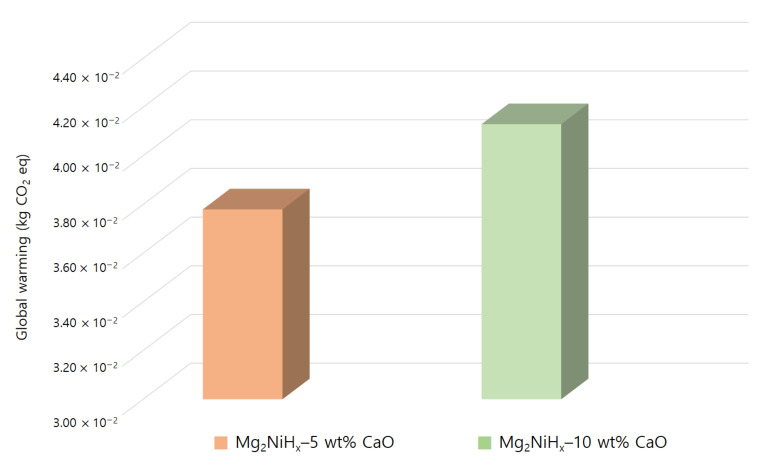
Comparing CO_2_ value of global warming impact by Mg_2_NiH_x_–CaO composites.

Figure 8 shows the CO_2_ value of global warming impact by synthesized Mg_2_NiH_x_–CaO composites. The carbon dioxide emission of Mg_2_NiH_x_–5 wt% CaO [28] was 0.0378 kg, Mg_2_NiH_x_–10 wt% CaO was 0.0413 kg. Accordingly, it was found that Mg_2_NiH_x_–10 wt% CaO with a higher CaO content had higher CO_2_ emissions than Mg_2_NiH_x_–5 wt% CaO. In order to decrease the occurrence of such global warming, various efforts for sustainable development have been reported in developed countries around the world to prevent environmental pollution by reducing CO_2_ emissions [29]. Therefore, it is necessary to improve the manufacturing process of the Mg_2_NiH_x_–CaO composites and to find the optimum mass ratio that can reduce the amount of CaO and increase the efficiency.

## 4. Conclusions

In this study, environmental pollution caused by the amount of CaO added during the synthesis of Mg_2_NiH_x_–CaO composites was evaluated through the LCA process. We generated 11 impact categories assessed using the EI99 methodology and 10 impact categories assessed using the CML 2001 methodology. As a result of the CML 2001 methodology, Mg_2_NiH_x_–5, 10 wt% CaO had the highest GWP value, and the resulting carbon dioxide generation was 0.0378 kg (Mg_2_NiH_x_–5 wt% CaO), 0.0413 kg (Mg_2_NiH_x_–10 wt% CaO). In addition, it can be seen that all environmental load values were overall higher depending on the amount of CaO added. Accordingly, in order to lower the global warming potential, it is necessary to save electricity or to find a more environmentally friendly material than CaO. The EI99 methodology showed the highest levels of fossil fuels, followed by climate change, followed by respiratory. This seems to be closely related to the aforementioned global warming, and the remaining environmental load values are very low and show similar results, so it is judged that the amount of CaO added did not have a significant effect. Therefore, efforts to reduce electricity usage as much as possible are expected to minimize unnecessary energy consumption in the synthesis process and to reduce large amounts of environmental impact figures. Ultimately, when comparing Mg_2_NiH_x_–5, 10 wt% CaO composites, Mg_2_NiH_x_–10 wt% CaO was better in terms of hydrogenation kinetics, but Mg_2_NiH_x_–5 wt% CaO showed better results in terms of environment. Therefore, it can be seen that the hydrogenation kinetics and the environmental load value are inversely proportional depending on the amount of CaO added. Ultimately, it is necessary to explore materials that exhibit excellent performance while using eco-friendly materials, and research should be conducted considering environmental factors, not performance-oriented, when making out an alloy.

## Figures and Tables

**Table 1:** Environmental impact categories applied using CML 2001.

Environmental Impact Categories	Unit	Life Cycle Environmental Impacts	
		Mg_2_NiH_x_–5 wt% CaO	Mg_2_NiH_x_–10 wt% CaO
Abiotic Resource Depletion (ARD)	Kg yr^−1^	6.85 × 10^−3^	8.76 × 10^−3^
Global Warming Potential (GWP)	Kg CO_2_ eq	3.86 × 10^−2^	4.09 × 10^−2^
Stratospheric Ozone Depletion Potential (ODP)	Kg CFC^−11^ eq	1.69 × 10^−5^	2.57 × 10^−5^
Photochemical Oxidation Potential (POCP)	Kg C_2_H_4_ eq	1.41 × 10^−5^	3.64 × 10^−5^
Acidification Potential (ACP)	Kg SO_2_ eq	1.83 × 10^−2^	1.45 × 10^−2^
Eutrophication Potential (EUP)	Kg PO_4_ eq	5.14 × 10^−3^	5.69 × 10^−3^
Fresh-water Aquatic Ecotoxicity Potential (FAETP)	Kg 1,4-DCB eq	1.57 × 10^−8^	2.25 × 10^−8^
Marine Aquatic Ecotoxicity Potential (MAETP)	Kg 1,4-DCB eq	1.89 × 10^−8^	3.17 × 10^−8^
Terrestrial Ecotoxicity Potential (TETP)	Kg 1,4-DCB eq	3.14 × 10^−4^	5.33 × 10^−4^
Human Toxicity Potential (HTP)	Kg 1,4-DCB eq	7.25 × 10^−8^	7.74 × 10^−8^

**Table 2:** Environmental impact categories Eco-Indicator 99’ (EI99).

Damage Categories		Damage Unit	Life Cycle Environmental Impacts	
			Mg_2_NiH_x_–5 wt% CaO	Mg_2_NiH_x_–10 wt% CaO
Human health	Carcinogenic effect	DALY	1.12 × 10^−8^	1.63 × 10^−8^
	Respiratory (organic)	DALY	2.47 × 10^−8^	2.68 × 10^−8^
	Respiratory (inorganic)	DALY	2.98 × 10^−2^	5.02 × 10^−2^
	Climate change	DALY	1.16 × 10^−1^	1.48 × 10^−1^
	Ionizing radiation	DALY	2.03 × 10^−2^	2.15 × 10^−2^
	Ozone depletion	DALY	1.26 × 10^−8^	1.29 × 10^−8^
Ecosystem quality	Ecotoxicity	PDF × m^2^ × yr	1.32 × 10^−2^	2.11 × 10^−2^
	Acidification/Eutrophication	PDF × m^2^ × yr	1.84 × 10^−2^	1.95 × 10^−2^
	Land-use	PDF × m^2^ × yr	1.16 × 10^−2^	1.19 × 10^−2^
Resources	Minerals	MJ	1.73 × 10^−2^	1.82 × 10^−2^
	Fossil	MJ	2.71 × 10^−1^	3.18 × 10^−1^

## Data Availability

The data presented in this study are available on request from the corresponding author.

## Nomenclature

Eco-Indicator 99’	As a lifecycle impact assessment tool developed by Consultants B.V., designers can perform environmental assessments of products by calculating environmental mark scores for used materials and processes.
ISO 14000 series	Environmental management life cycle assessment principles and framework, Korean Agency for Technology and Standard, 2007.
Global warming potential (GWP)	In a relative sense, it is a type of index based on a simplified radiative characteristic that can be used to measure the possible future impacts of the evolution of various gases on the climate system in the future.
ODP (ozone layer depletion potential)	It is a numerical expression of the degree of destruction of compounds that destroy ozone. Based on the ozone depletion capacity of CFC-11 as 1, the destructive power of the remaining chemicals was assumed.
AP (acidification potential)	Acidification occurs primarily when nitrogen oxides (NO_x_) and sulfur dioxide gas (SO_2_) interact with other atmospheric components.
EP (eutrophication potential)	It refers to a phenomenon in which nutrients are excessively supplied to water due to the inflow of chemical fertilizers or sewage, causing rapid growth or extinction of plants and killing organisms by taking away oxygen from the water.
ETP (ecotoxicity potential)	Ecotoxicity refers to the ecological impacts of chemicals, pesticides, and pharmaceuticals on freshwater organisms and the possible risks to the aquatic ecosystem.
Human toxicity potential (HTP)	Total emissions are assessed in terms of benzene and toluene equivalents, but potential doses include multiple routes of exposure, including inhalation, ingestion, and dermal contact of fish and meat.

## References

[1] Ryi S.K., Han J.Y., Kim C.H., Lim H., Jung H.Y. (2017). Technical Trends of Hydrogen Production. Clean Technol..

[2] Balat M. (2008). Potential Importance of Hydrogen as a Future Solution to Environmental and Transportation Problems. Int. J. Hydrogen Energy.

[3] Demirbas A., Dincer K. (2008). Sustainable Green Diesel: A Futuristic View. Energy Sources Part A.

[4] Yun C.W. (2019). Liquid Organic Hydrogen Carrier (LOHC) technology for realization of a hydrogen society. News Inf. Chem. Eng..

[5] Lee P.J., Kim J.W., Bae K.K., Jeong S.U., Kang K.S., Jung K.J., Park C.S., Kim Y.H. (2020). Heat Transfer Characteristics and Hydrogen Storage Kinetics of Metal Hydride-Expended Graphite Composite. Trans. Korean Hydrog. New Energy Soc..

[6] Zhi C., Chao T., Hui P., Huabin Y. (2010). Rehydrogenation performance of an MgH_2_–Nb_2_O_5_ system modified by heptane and acetone. Int. J. Hydrogen Energy.

[7] Yang W.N., Shang C.X., Guo Z.X. (2010). Site density effect of Ni particles on hydrogen desorption of MgH_2_. Int. J. Hydrogen Energy.

[8] Park H.R., Song M.Y. (2017). Reaction Rate with Hydrogen and Hydrogen-storage Capacity of an 80Mg+14Ni+6TaF5 Alloy Prepared by High-energy Ball Milling in Hydrogen. Trans. Korean Hydrog. New Energy Soc..

[9] Jang S.Y., Kang K.M., Hayato O., Shigeharu K. (2003). Mg-based hydrogen storage alloy. Gas Ind. Technol..

[10] Berlin J. (2002). Environmental life cycle assessment (LCA) of Swedish semi-hard cheese. Int. Dairy J..

[11] Lee S.H., Jo Y.M. (2010). Review of National Policies on the Utilization of Waste Metal Resources. KIC News.

[12] Lee S.S., Hong T.W. (2014). Life Cycle Assessment for Proton Conducting Ceramics Synthesized by the Sol-Gel Process. Materials.

[13] Rhys J.T., Marco L.L., Andrew N., Kevin D.P., Ian H. (2020). An evaluation of life cycle assessment and its application to the closed-loop recycling of carbon fibre reinforced polymers. Compos. Part B Eng..

[14] Lee N.R., Lee S.S., Kim K.I., Hong T.W. (2012). Environmental Assessment of Chemically Strengthened Glass for Touch Screen Panel by Material Life Cycle Assessment. Clean Technol..

[15] Lee S.S., Lee N.R., Kim K.I., Hong S.J., Hong T.W. (2012). MLCA (Material Life Cycle Assessment) for ITO Recycling. Mater. Sci. Forum..

[16] Hong T.W., Lim J.W., Kim S.K., Kim Y.J., Park H.S. (1999). Formation of Mg_2_NiH_x_ hydrogen absorbing materials by hydrogen induced mechanical alloying. J. Korean Inst. Met. Mater..

[17] Yasin S., Behary N., Perwuelz A., Guan J. (2018). Life cycle assessment of flame retardant cotton textiles with optimized end-of-life phase. J. Clean. Prod..

[18] International Organisation for Standardisation (2006). ISO 14040: 2006—Environmental Management—Life Cycle Assessment—Principles and Framework.

[19] International Organisation for Standardisation (2006). ISO 14044: 2006—Environmental Management—Life Cycle Assessment—Requirements and Guidelines.

[20] Meng F., McKechnie J., Turner T.A., Pickering S.J. (2017). Energy and environmental assessment and reuse of fluidised bed recycled carbon fibres. Compos. Part A Appl. Sci. Manuf..

[21] Demertzi M., Silvestre J.D., Durão V. (2020). Life cycle assessment of the production of composite sandwich panels for structural floor’s rehabilitation. Eng. Struct..

[22] Jeong S.J., Lee J.Y., Shon J.S., Hur T. (2006). Life Cycle Assessments of Long-term and Short-term Environmental Impacts for the Incineration of Spent Li-ion Batteries (LIBs). J. Korean Ind. Eng. Chem..

[23] Shin H.W., Hwang J.H., Kim E.A., Hong T.W. Hydriding Kinetics on Mg_2_NiH_x_-5wt% CaO Composites. Trans. Korean Hydrog. New Energy Soc..

[24] Lee J.K., Kim S.K. (2011). Effect of CaO addition on the ignition resistance of Mg-Al alloys. Mater. Trans..

[25] Huang Z.G., Guo Z.P., Calka A., Wexler D., Lukey C., Liu H.K. (2006). Effects of iron oxide (Fe_2_O_3_, Fe_3_O_4_) on hydrogen storage properties of Mg-based composites. J. Alloys Compd..

[26] Sahoo S.K., Parveen S., Panda J.J. (2007). The present and future of nanotechnology in human health care. Nanomed. Nano Technol. Biol. Med..

[27] Rashidi A.M., Nouralishahi A., Khodadadi A.A., Mortazzavi Y., Karimi A., Kashefi K. (2010). Modification of single wall carbon nanotubes (SWNT) for hydrogen storage. Int. J. Hydrogen Energy.

[28] Shin H.W., Hwang J.H., Kim E.A., Hong T.W. Material Life Cycle Assessment on Mg_2_NiH_x_-5 wt% CaO Hydrogen Storage Composites. Clean. Technol..

[29] Kim M.G., Son J.T., Hong T.W. (2018). Evaluation of TiN-Zr Hydrogen Permeation Membrane by MLCA (Material Life Cycle Assessment). Clean. Technol..

